# Snakebite Envenoming Diagnosis and Diagnostics

**DOI:** 10.3389/fimmu.2021.661457

**Published:** 2021-04-28

**Authors:** Cecilie Knudsen, Jonas A. Jürgensen, Sofie Føns, Aleksander M. Haack, Rasmus U. W. Friis, Søren H. Dam, Sean P. Bush, Julian White, Andreas H. Laustsen

**Affiliations:** ^1^ Department of Biotechnology and Biomedicine, Technical University of Denmark, Kongens Lyngby, Denmark; ^2^ BioPorto Diagnostics A/S, Hellerup, Denmark; ^3^ Department of Emergency Medicine, Yale School of Medicine, New Haven, CT, United States; ^4^ Toxinology Department, Women’s and Children’s Hospital, North Adelaide, SA, Australia

**Keywords:** envenoming, clinical toxinology, diagnosis, diagnostics, ophidism, snakebite management, syndromic approach

## Abstract

Snakebite envenoming is predominantly an occupational disease of the rural tropics, causing death or permanent disability to hundreds of thousands of victims annually. The diagnosis of snakebite envenoming is commonly based on a combination of patient history and a syndromic approach. However, the availability of auxiliary diagnostic tests at the disposal of the clinicians vary from country to country, and the level of experience within snakebite diagnosis and intervention may be quite different for clinicians from different hospitals. As such, achieving timely diagnosis, and thus treatment, is a challenge faced by treating personnel around the globe. For years, much effort has gone into developing novel diagnostics to support diagnosis of snakebite victims, especially in rural areas of the tropics. Gaining access to affordable and rapid diagnostics could potentially facilitate more favorable patient outcomes due to early and appropriate treatment. This review aims to highlight regional differences in epidemiology and clinical snakebite management on a global scale, including an overview of the past and ongoing research efforts within snakebite diagnostics. Finally, the review is rounded off with a discussion on design considerations and potential benefits of novel snakebite diagnostics.

## Introduction

Every year, people lose their livelihoods, limbs, and lives to a disease that is as neglected as it is ancient: Snakebite envenoming. The exact burden of snakebite envenoming is notoriously difficult to assess, because data on envenoming prevalence are scarce, and the available data points are often inaccurate or not representative for broader geographical areas (1–4). Nonetheless, studies suggest that mortality due to snakebite envenoming may exceed 125,000 deaths per year globally, while the number of people suffering permanent sequelae may be around 400,000, and the toll of associated disability-adjusted life years might add up to a total of over 6 million (4–8). To make matters worse, snakebite envenoming is both a disease mainly affecting the poor and a disease that leads to further impoverishment (7, 9–12). In spite of the immense burden of snakebite envenoming on victims, their families, and local communities, the disease remains largely neglected and has historically only received few resources, and limited efforts have gone into the development of better treatments and diagnostics (13).

Once diagnosed, the mainstay treatment of severe envenoming is antivenom in combination with auxiliary treatment (7, 14, 15). Monovalent antivenoms can be used when the species of the offending snake is known, while polyvalent antivenoms are useful in cases where the snake species has not been identified. However, the ability of polyvalent antivenoms to neutralize a broad range of venoms might come at the cost of decreased efficacy, as the relative proportion of antibodies in a polyvalent antivenom that targets toxins of a specific snake venom is often not as great as the proportion of antibodies in a monovalent antivenom targeting the same venom toxins. Therefore, it can become necessary to administer a greater dose of a polyvalent antivenom than of a monovalent antivenom in order to treat a given envenoming (7, 16). Increasing the dose can, in turn, affect the cost of treatment and the risk of developing adverse reactions to the antivenom (7). Unfortunately, polyvalent antivenoms are favored in many places either due to the lack of monovalent antivenoms or due to the difficulty in choosing which monovalent antivenom to administer in the absence of reliable information on the perpetrating snake species (17).

In addition to enabling the administration of monovalent antivenoms, where available, identifying the offending snake species or the type of venom might enable clinicians to predict and prepare for the development of clinical manifestations. To aid clinicians in this task, there is a common and deceptively simple categorization of elapid venoms as being primarily neurotoxic and viperid venoms as being primarily cytotoxic and/or hemotoxic (here understood as toxicity directed toward blood and the cardiovascular system, including hemostasis) (7). While this simplification does represent a general trend, it can cause clinical misinterpretation and there are several important exceptions to the rule. For example, many major Australian elapid snakes commonly cause coagulopathy, often without evident neurotoxicity, while some important viperids cause minimal cytotoxicity, yet important neurotoxicity (18). Similarly, bites from some elapid species of the cobra genus, particularly spitting cobras, can cause strong cytotoxic symptoms without causing neurotoxicity (19), which may be confused with viper envenomings. Matters are further complicated by the fact that snake venom composition can vary within genera and even within species due to ontogeny and geographical distribution (20–23). As such, the variability of clinical manifestations of envenoming and the time courses of their development complicate the treatment of snakebite (7). Thus, while snakebite is generally well-handled in many areas, room for innovation and improvement still exists. Encouragingly, recent years have seen a renewed interest in such innovations and improvements, with much research being published not only on novel treatment modalities (e.g. recombinant antivenoms and small molecule inhibitors) but also on novel diagnostics (e.g. enzyme-linked immunosorbent assays, lateral flow assays, impedimetric immunoassays, infrared imaging, and polymerase chain reaction-based methods) [see **Table 1** and (104–108)]. In time, some of these diagnostic tools may enter the clinic, where they could be utilized to obtain valuable information, such as the identity of the perpetrating snake species or genus, allowing use of monovalent antivenoms, or quantitative measures of the degree of envenoming. Additionally, if implemented in rural settings, diagnostic kits may guide treatment decision for less experienced clinicians, enabling proper management of snakebite victims at rural facilities. This information might support clinical management of snakebite envenoming and epidemiological studies of relevance to antivenom development, resource management, and advocacy for increased attention to snakebite.

**Table 1:** Overview of snakebite diagnostics capable of differentiating snake venoms.

Type	Subtype	Abs	Area	Snake(s) targeted	LoD	Assay duration	Tested on patient samples?	Sample matrix	Notes	Year	Ref.
Immuno-assay	Immuno-diffusion	Equine		Cobra spp. (possibly *O. hannah*)	1:100,000,000 dilution		Patients: 1 (case study)	Tissue homogenate		1957	(24)
	Immuno-diffusion	?	Australia	*A. antarcticus*, *N. scutatus*, *P. porphyriacus*, *P. textilis*	?	>3 hours	?	Serum exudate (guineapig)	Only abstract available	1967	(25)
	Agglutination test	Caprine & equine	California	*C. v. helleri*		>2 hours	Patients: 16	Serum		1968	(26)
	Immuno-diffusion	Leporine	Africa	*B. arietans*, *C. maculatus*, *E. carinatus*, *N. haje*, *N. melanoleuca*, *N. nigricollis*		48 hours (immuno-difussion) & >1 hour (immuno-electrophoresis)	Patients: 101	Wound aspirates, blister fluids, sera, urine	Sensitivity: 39.6% (40/101)	1974	(27)
	RIA		Australia	*N. scutatus*, *P. textilis*	<10 ng	>24 hours	Patients: 2 (also tested on rabbit serum)	Serum, sample buffer		1974	(28)
	RIA		Australia	*N. scutatus*, *P. textilis*		>24 hours	Patients: 3	Tissue samples, fluids		1975	(29)
	ELISA	Leporine & equine IgGs	Multiple	*B. arietans*, *C. maculatus*, *E. carinatus*	1-5 ng/mL	O/N incubation	No – Tested in rodents	Serum (human and rat)	Cross-reactivity to the following species tested: *A. rhodostoma, B. gabonica, C. adamanteus, E. schistosa, N. haje, N. naja, N. nigricollis, O. scutellatus, V. berus*	1977	(30)
	RIA	Leporine	Australia	*N. scutatus*		O/N incubation	Patients: 3	Urine, serum, clothing, tissue samples		1977	(31, 32)
	RIA	Leporine IgG	Australia	*A. antarcticus*, *A. superba*, *N. scutatus*, *O. scutellatus*, *P. australis*, *P. porphyriacus*, *P. textilis*	0.1-0.4 ng/mL	>20 hours	Unpublished	Tissue samples		1978	(33)
	ELISA	Leporine IgG		?	0.5-2 ng/mL	30-90 minutes	?	?	Only abstract available	1980	(34)
	Enzyme immunoassay	Leporine IgG	Australia	*Acantophis* spp., *Notechis* spp., *Oxyuranus* spp., *Pseudoechis* spp., *Pseudonaja* spp.	5-15 ng/mL	20-40 minutes	Patients: 43	Whole blood, urine, wound swab		1982	(35)
	ELISA	?	?	?	1 ng/mL	>3 hours	No - Tested in rabbits	Blood, urine, exudate	Only abstract available	1983	(36)
	ELISA	Leporine IgG	?	*A. rhodostoma*, *N. naja*	7.8-15.6 ng/mL	35-45 minutes	Yes	Serum	Only abstract available	1983	(37)
	ELISA	Leporine IgG	USA	*A. contortrix*, *C. atrox*, *C. scutulatus*	0.1-.01 µg/mL	O/N incubation	No - Tested in animals	Sero-sanguineous fluid, blood, urine, peritoneal fluid, pleural fluid, lung, kidney	Cross-reactions with venoms of other snakes were extensive at higher concentrations	1984	(38)
	ELISA	Equine	Myanmar	*D. russelii*	10 ng/mL	O/N incubation	Yes	Serum	No cross-reactivity found to *B. fasciatus, N. naja, O. hannah, T. gramineus*	1984	(39)
	ELISA	Leporine	Thailand	*N. kaouthia*	2 ng/well		Yes	Serum		1986	(40)
	Enzyme-linked coagulation assay	Murine monoclonal IgG		*D. russelii*	2-10 pg/mL		No			1987	(41)
	ELISA		Nigeria	*B. arietans*, *C. maculatus*, *E. carinatus*		O/N incubation	Patients: 31	Blood, serum, urine, sputum, bite site aspirates		1987	(42)
	ELISA		Philippines	*N. n. philippinensis*		O/N incubation	Patients: 1 (postmortem)	Blood		1987	(43)
	ELISA	Leporine IgG	Thailand	*C. rhodostoma*, *D. russelii*, *N. kaouthia*, *T. albolabris*	10-20 ng/mL	O/N incubation	Patients: 251	Serum		1987	(44)
	RIA	Murine monoclonal Abs	Thailand	*D. russelii*	4-20 ng/mL depending on matrix	O/N incubation	Patients: 4	Serum, urine	Known to cross-react with cobra venom.	1987	(45)
	ELISA	Equine	Europe	*V. ammodytes*	<1 ng/mL	<20 minutes	No - Tested in rabbits	Blood	Specificity mentioned as being a problem.	1988	(46)
	ELISA	?	Asia	*A. b. blomhoffii*	5.4 ng/well	?	No - Tested in mice	Serum	Only abstract available. No cross-reactivity to *R. t. tigrinus* venom.	1988	(47)
	ELISA	Leporine IgG	Brazil	*B. jararaca*	14.6 ng/mL	O/N incubation	No – Tested in mice	Serum	Tested for cross-reactivity to *Bothrops* spp., *Crotalus* spp., *Lachesis* spp., and *Tityus serrulatus* venom.	1990	(48)
	ELISA	?	?	*A. b. blomhoffii*	?	?	No - Tested in rats & rabbits	Serum	Only abstract available	1990	(49)
	Agglutination test	Leporine IgG	Thailand	*B. fasciatus*, *C. rhodostoma*, *D. russelii*, *N. kaouthia*, *N. n. siamensis*, *O. hannah*, *T. albolabris*	0.16-1.2 µg/mL	40 minutes	Serum samples: 59 Wound swabs: 26	Serum, wound swabs	Sensitivity of 52.5%. Tested for hook effect and interference from sample matrices.	1991	(50)
	ELISA	Equine F(ab’)_2_	Brazil	*C. d. terrificus*	1-3 pg/mL	O/N incubation	No – Tested in mice	Serum (mice), sample buffer		1991	(51)
	ELISA	Leporine IgG	Myanmar	*D. russelii*	10 ng/mL	O/N incubation	Patients: 311 Controls: 118	Serum	Specificity 88% (14 false positives from 118 negatives). Tested for cross-reactivity to *B. fasciatus, N. kaouthia, O. hannah, T. erythrurus*.	1991	(52)
	ELISA	Equine F(ab’)_2_	Europe	*Vipera* spp.	2-7 ng/mL (depending on sample matrix)	>4.5 hours	Yes	Blood, serum, urine	Tested for cross-reactivity to *B. jararaca* and *C. d. terrificus*.	1992	(53–56)
	ELISA	Leporine IgG	Australia	*A. antarcticus*, *N. scutatus*, *O. scutellatus*, *P. australis*, *P. textilis.*	2.5 ng/mL	O/N incubation	Unpublished	Sample buffer		1992	(57)
		Leporine IgG	Southern Thailand	*C. rhodostoma*	5 ng/mL	50 minutes	No	Sample buffer	Cross-reactivity to 26 venoms tested.	1992	(58)
	Agglutination test	Leporine IgG	Thailand	*B. fasciatus*, *C. rhodostoma*, *D. russellli*, *N. kaouthia*, *O. hannah*, *T. albolabris*	2-635 ng/mL	60-120 minutes	Serum samples: 59 Wound swabs: 26	Serum, wound swab	Sensitivity of 81.3% for serum samples and 61.5% for wound swabs. Cross reactivity at concentrations at least 62 times higher.	1993	(59)
	ELISA	Leporine IgG	Brazil	*B. alternatus*, *B. atrox*, *B. jararaca*, *B.jararacussu*, *B. moojeni*, *B. neuwedi*, *C. d. terrificus*, *C. d. collineatus*, *L. muta*	<0.01-0.1 µg/mL	O/N incubation	No	Sample buffer, serum (non-envenomed humans)		1993	(60)
	ELISA	Leporine IgG	Brazil	*B. atrox*, *L. m. muta*	20 ng/mL	2 hours	Yes	Serum	Also tested in mice.	1993	(61)
	Fluorogenic ELISA	?		*D. russelii*	0.1 pg/mL	?	?	?	Only abstract available. Shown to cross-react with several other venoms.	1993	(62)
	ELISA		Papua New Guinea	*P. papuanus*		O/N incubation	Patients: 9	Serum, urine, wound aspirates		1994	(63)
	ELISA	Leporine IgG	Tunisia	*E. pyramidum*	<10 ng/mL	O/N	Yes	Serum		1994	(64)
	ELISA		North America	*Agkistrodon* spp.	2 ng/mL	>5 hours	No – Tested in rabbits	Serum		1994	(65)
	ELISA		Myanmar	*O. hannah*	<20 ng/mL		Patients: 2 (case studies)	Serum		1995	(66)
	ELISA & RIA	Ovine Fab	Europe	*Vipera* spp.	0.8 ng/mL (ELISA) & 2 ng/mL (RIA)	>3 hours (ELISA) & O/N incubation (RIA)	Yes	Plasma, urine		1996	(67)
	ELISA	Leporine F(ab’)_2_	India	*B. caerulus*, *D. russelii*, *E. carinatus*, *N. naja*	1 ng/mL	30 min	Patients: 27	Blood, serum, urine, wound swab	Only abstract available	1996	(68)
	ELISA	Equine F(ab’)_2_	Martinique	*B. lanceolatus*		3 hours	Patients: 40 Controls: 120	Serum	Sensitivity 46%, specificity 88%.	1997	(69)
	ELISA & agglutination assay	Equine	Central America	*Micrurus* spp.	0.3 mg/mL (agglutination assay) & 4 ng/mL (ELISA)	>5 minutes (agglutination test) & O/N incubation (ELISA)	No – Tested in rabbits and mice	Serum, plasma		1997	(70)
	ELISA	Caprine & leporine IgG	India	*Bungarus* spp., *Daboia* spp., *Echis* spp., *Naja* spp.	0.1 ng/mL	>5 hours	Yes (postmortem only)	Tissue samples		1999	(71)
	ELISA	?	Taiwan	Cobra spp.	0.5 ng/mL	6 hours	?	Calf serum and human urine	Only abstract available	2002	(72)
	Optical immunoassay	Leporine IgG	Asia	*B. multicinctus*	2.5-10 ng/mL	25 minutes	No – Tested in mice	Blood, tissue samples	Cross-reactivity to 11 venoms and toxins tested.	2002	(73)
	Agglutination test	Equine	Venezuela	*Bothrops* spp., *Crotalus* spp.	167 µg/mL	10 minutes	No	Sample buffer	LoD unit uncertain.	2004	(74)
	Optical immunoassay	Leporine IgG	Vietnam	*C. rhodostoma*, *N. kaouthia*, *O. hannah*, *T. albolabris*	0.2-0.8 ng/mL depending on the venom and sample matrix	40 minutes	Patients: 83 Samples: 125	Serum, urine, wound exudate		2004	(75)
	ELISA	Avian IgY & leporine IgG	India	*N. naja*	0.1-300 ng	O/N incubation	Patients: 12 (live) Patients: 7 (postmortem)	Skin, blood, cerebrospinal fluid		2006	(76)
	ELISA	Leporine IgG	India	*B. caeruleus*, *N. naja*		O/N incubation	Samples: 22 (postmortem)	Skin, blood		2007	(77)
	Immuno-flourescence			*N. kaouthia*	5–10 ng/mL	3 hours	No	Sample buffer		2008	(78)
	ELISA	Leporine IgG	Australasia	*Oxyuranus* spp.	0.15 ng/mL	O/N incubation	Patients: 17	Serum	Also tested in rat serum and for cross-reactivity with Australian snake venoms.	2010	(79)
	ELISA	Leporine IgG	Colombia	*L. acrochorda*	3.9 ng/mL		No	Sample buffer	Specificity 100%.	2012	(80)
	ELISA	Leporine IgG	Egypt	*N. haje*, *N. nigricollis*, *W. aegyptia*	<10 ng/well	O/N incubation	No	Sample buffer	Avidities of 2.5-2.8 depending on the venom	2013	(81)
	LFA	Avian	Taiwan	*N. atra*	5 ng/mL	20 minutes	Patients: 88 (34 cobra and 54 non-cobra)	Serum	Sensitivity 83.3%, specificity 100%.	2014	(82)
	LFA	Equine & leporine IgG	India	*Daboia* spp.*, Naja* spp.	0.1 ng/mL	10 minutes	No - Tested in mice	Plasma		2016	(83)
	ELISA	Leporine IgG	India	*Bungarus* spp., *Daboia* spp., *Echis* spp., *Naja* spp.	1 ng/mL	20-25 minutes	No - Tested in mice	Sample buffer		2017	(84)
	ELISA & LFA	Equine	Taiwan	Neurotoxic vs hemorrhagic venom	LoD of 5-50 ng/mL (LFA) & LoQ of 0.39-0.78 ng/mL (ELISA)	10-15 minutes	Patients: 21	Serum	Sensitivity and specificity of 100% for neurotoxic venoms. Sensitivity of 36.4% for hemorrhagic venoms.	2018	(85)
	Impedimetric immunoassay	Equine	Brazil	*Bothrops* spp.	0.27 ug/mL	>25 minutes?	No	Sample buffer	Tested for cross-reactivity to *C. d. terrificus* and *M. leminiscatus.*	2018	(86)
	ELISA	Leporine	Sri Lanka	*B. caeruleus*, *D. russelii*, *H. hypnale*, *N. naja*	0.19-1.56 ng/mL (depending on the venom)	>2 hours	Patients: 19 Controls: 20	Serum	Quantitative. Cross-reactivity between the venoms was tested.	2020	(87)
	LFA	Avian & equine	South-East Asia	*Daboia* spp., *Naja* spp.	10 ng/mL (*in vitro*)	25-30 minutes	Samples: 5	Serum		2020	(88)
	LFA	Leporine & equine	Asia & Africa	*Naja* spp.	5-10 ng/mL for Asian cobras and <500 ng/mL for African cobras	>20 minutes	No	Serum (fetal bovine)	Based on (82).	2020	(89)
Molecular biology	PCR	N/A	Thailand	*N. kaouthia*		>2 hours	No - Tested on mice	Wound swabs (mice)	Also tested on venom from *B. fasciatus, C. rhadostma, D. russelii, O. Hannah.*	2001	(90)
	PCR	N/A	Thailand	*B. fasciatus*, *C. rhodostoma*, *D. siamensis*, *Hydrophiinae* spp., *Naja* spp., *O. Hannah*, *Trimeresurus* spp.	0.025 ng/mL	>67 minutes	No	Saliva (snake)		2015	(91)
	PCR	N/A	Nepal	*Bungarus* spp., *Naja* spp., *O. hannah*, *O. moticola*, *Trimeresurus* spp.		O/N incubation	Patients: 565	Wound swab	Specificity 100%.	2016	(92)
Misc.	Enzymatic activity assay	N/A	Sri Lanka & Australia	*B. caeruleus*, *D. russelii*, *H. hypnale*, *N. naja*, *P. porphyriacus*			Patients: 108	Serum		2014	(93)
	Infrared thermography	N/A	Brazil	*B. moojeni*, *C. d. terrificus*, *B. jararaca*		>15 minutes	Patients: 8			2017	(94)
	Enzymatic activity assay & ELISA	N/A	Australia	Elapid spp.	0.1-0.2 ng/mL (ELISA)		Patients: 115 Controls: 80	Serum		2018	(95)

Many studies did not report on the duration of the diagnostic procedure. In such cases, assay duration was reported in this table as “> total incubation time”, e.g. “>3 hours” for an assay with an incubation time of 3 hours. For other unreported values, the corresponding fields were left empty. In some cases, only the abstracts of the studies were available to us, and in these cases, values not reported in the abstract have been marked “?”. Studies describing the detection of venom-specific antibodies in snakebite victims [e.g. (96–98)] or the detection of toxins or toxin activities for the purpose of venom characterization rather than diagnosis [e.g. (99–103)] were not included in this table. Abs, antibodies; ELISA, enzyme-linked immunosorbent assay; F(ab’)_2_, fragment antigen binding 2; IgG, immunoglobulin G; LFA, lateral flow assay; O/N, overnight; PCR, polymerase chain reaction; RIA, radioimmunoassay.

## Commonly Adopted Approaches for Diagnosis of Snakebite Envenoming

A basic diagnosis of snakebite envenoming requires a thorough patient history, targeted examination, and appropriate laboratory investigations (109). Taking a detailed history includes asking about the circumstances of the bite (e.g. geography, time of the incident, activity, and number of bites), details of the snake (if seen, brought, or photographed), clinical manifestations of envenoming (including time of onset), first aid applied, and past medical history (e.g. allergies, prior snakebites, relevant medications, and pre-existing medical conditions) (109). Laboratory investigations almost always include an evaluation of the blood clotting profile to screen for venom-induced coagulopathies. In its simplest form, a blood clotting test can be carried out in the form of a 20 minute whole blood clotting test (20WBCT). If more sophisticated equipment is available, it is common to run repeated tests of the International Normalized Ratio (INR) of blood clotting, activated partial thromboplastine time (aPTT), D-dimer, and/or fibrinogen degradation products (FDP), supplemented by hemograms and potentially also by electrocardiograms. Acute falls in hemoglobin and hematocrit values may indicate internal bleeding, and a drop in fibrinogen levels might be indicative of coagulopathy (7, 110, 111). Blood samples are usually also screened for creatine kinase (CK) levels, electrolytes, urea, nitrogen/creatinine, which together with urinalysis (hematuria, proteinuria, urea levels, and urine output) can be used to assess venom-induced rhabdomyolysis and associated complications, such as myoglobinuric renal failure or polyuria, oliguria, or anuria (110, 111). See **Table 2** for a list of these laboratory investigations. Based on the patient history and laboratory tests, trained toxinologists may be able to infer the offending snake species, and this can in turn guide the choice of treatment.

**Table 2:** Examples of auxilliary tests that are frequently performed for suspected snakebite victims.

Auxilliary tests	
Type	Subtype
Hemograms	Platelet count
	Blood count (hemoglobin, white cell count, absolute lymphocyte count)
	Examination of blood film for evidence of intravascular hemolysis (schistocytes, spherocytes, etc.)
Clotting profile	Fibrinogen level
	Prothrombin time/INR of blood clotting
	Activated partial thromboplastine time (aPTT)
	D-dimer/fibrinogen degradation products (FDP)
Serum biochemistry	Electrolytes
	Bilirubin
	Liver function tests
	Creatine kinase (CK; CPK)
Urinalysis	Hematuria
	Myoglobinuria
Renal function	Serum creatinine
	Urea
	Glomerular filtration rate
	Urine output (polyuria, oliguria, anuria)
Electrocardiagram	

Diagnostic algorithms summarize much of the knowledge required to diagnose snakebites. They have been developed for some settings and regions to provide support for doctors and other healthcare workers tasked with frontline management of patients with suspected envenoming. Here, it is acknowledged that frontline staffs will often have limited training in managing envenoming and limited rapid access to clinical toxinology expertise to guide their important treatment decisions in the critical early hours after a bite. The purpose of such diagnostic algorithms is to synthesize and distill the knowledge and experience of experts in clinical toxinology into a readily and rapidly accessible format to guide less experienced health professionals toward optimal care of bitten and envenomed patients. Formally assessing the effectiveness of diagnostic and treatment algorithms for envenoming is a challenge, with no clear published research available. However, experience in countries such as Australia (first world) and Myanmar (developing world) appears to indicate that diagnostic algorithms developed for snakebite, individualized for each country or region, are both widely used and accepted. In Myanmar, snakebite diagnostic algorithms were developed by a team of health professionals through a series of drafts, tested by frontline healthcare workers, and a final version was adopted and rolled out nationally by the Ministry of Health. Feedback from frontline healthcare workers in Myanmar was strongly positive. Diagnostic algorithms do not replace expertise in clinical toxinology, but can be an important part of an optimal care pathway. However, it must always be acknowledged in such algorithms that they are merely a guide and cannot cater for every possible clinical scenario and presentation.

As previously mentioned, it is also a common procedure to ask whether the patient saw the snake, and if so, what it looked like (112). However, using the victim’s description of the snake is often not, in isolation, a reliable diagnostic method for identifying the snake, although for some snakes in some countries (e.g. Russell’s viper in Myanmar) it may be reasonably reliable. In some cases, the biting snake is not seen, and even if it has been spotted, the victim’s description can potentially be misleading (113). While it may be easier to identify the snake if it is brought to the hospital for identification, even in these cases, misidentification can occur, and in some communities, there is an unwillingness among hospital staff to inspect or handle the snake (114). As an example, hump-nosed pit vipers (*H. hypnale*) are often misidentified as saw-scaled vipers (*E. carinatus*) in India, resulting in administration of ineffective antivenom (115). Even if the snake has been positively identified by an expert herpetologist, the clinical presentation of the patient is pivotal, as different specimens of venomous snakes (e.g. from different regions) can cause different clinical envenoming syndromes (109, 116). Another caveat of this approach is the inherent risk of attempting to capture or kill the snake, which can lead to further envenoming of the victim or a helper attempting to catch the snake; however, if a snake has already been killed, this is a potentially valuable diagnostic aid. Hospital staff should be encouraged to examine and preserve all such presented snakes, as this can allow retrospective studies clearly defining medically relevant species for a particular region. In most settings, 70% ethanol is an appropriate preservative for dead snakes, immersing the entire snake and injecting the preserving fluid into the body cavity. It is critically important that preserved snakes are adequately labeled and that preservation methods that will not lead to deterioration of the label (in the preserving fluid) are employed, so that subsequent examination can unequivocally link the dead snake to a particular snakebite patient.

## Snakebite Diagnosis in Australia

In Australia, snakebites are diagnosed based on patient history and laboratory investigations, as described above. In combination with neurological assessment, it is possible to identify most severe envenoming cases within 12 hours of the bite (117), after which patients with confirmed or suspected envenoming can be discharged if clinical findings and laboratory test results indicate no envenoming has occurred. In rare cases, envenoming – in particular by death adders – does not manifest itself before 24 hours post-bite, though it is unclear whether such late-presenting envenomings can progress to severe or life-threatening envenomings in subsequent hours.

**Figure 1:**
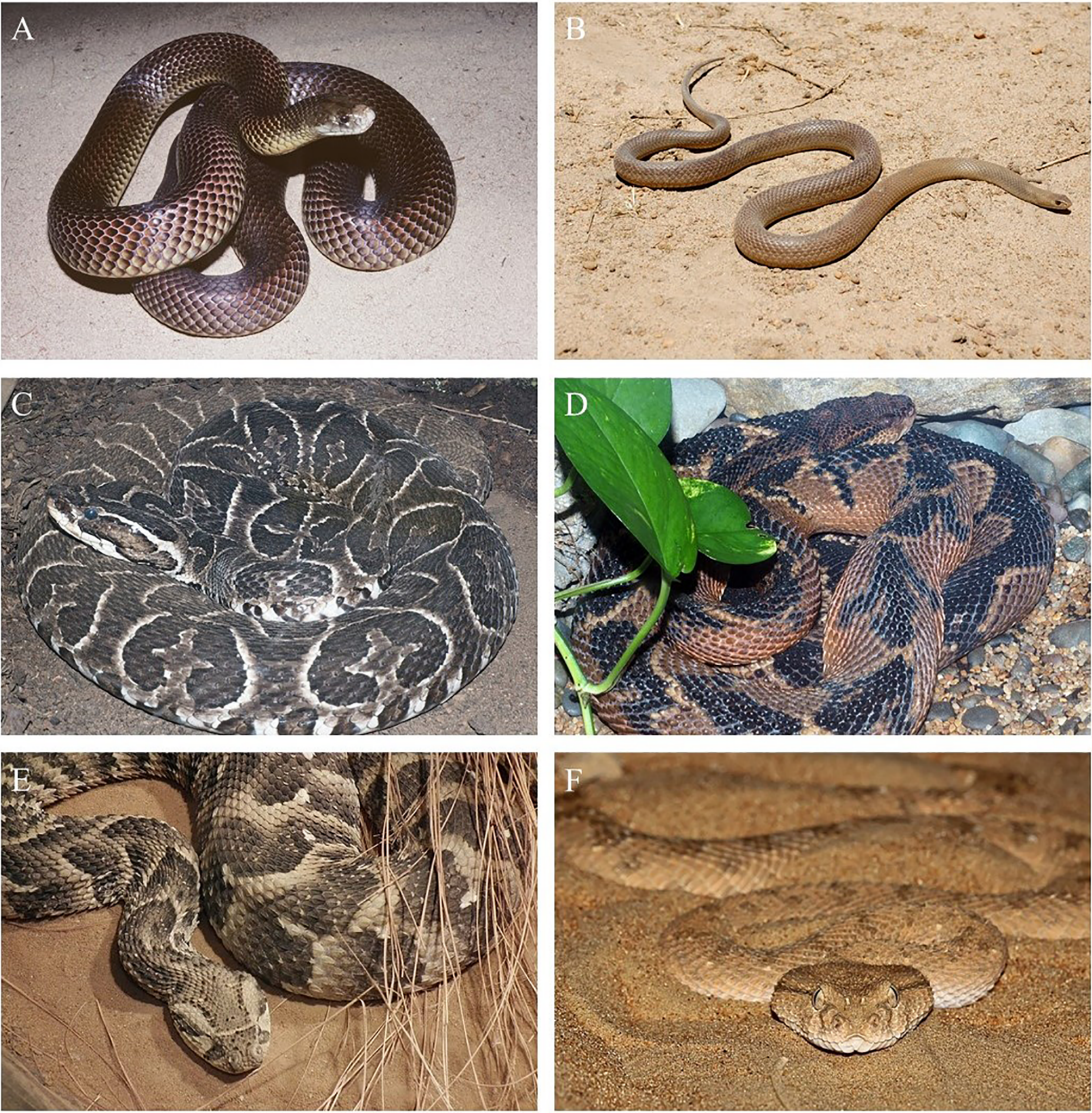
Comparison of venomous snakes with similar names and/or appearance and/or clinical syndromes. Visual comparison of two Australian snakes with similar names and appearances: **(A)** A king brown snake (*Pseudonaja textilis*), which belongs to the black snake genus, and **(B)** an eastern brown snake (*Pseudonaja textilis*), which belongs to the brown snake genus. Visual comparison of two venomous snake species from Brazil: **(C)** *Bothrops* sp. and **(D)** *Lachesis* sp. Species from these genera can appear similar to those not trained in snake identification, can cause similar clinical manifestations, and are both locally known as ‘surucucu’ in certain parts of Brazil. Visual comparison of **(E)** a puff adder (*Bitis arietans*) and **(F)** a horned viper (*Cerastes cerastes*), the venoms of which can cause similar clinical manifestations. **Figures 1A, C, D** copyright ^©^ Prof. Julian White, **Figure 1B** copyright ^©^ of Prof. Sean Bush, **1E, 1F** were found on WikiMedia Commons are are copyright ^©^ of the user 4028mdk09 and the user Broobas, respectively.

**Figure 2:**
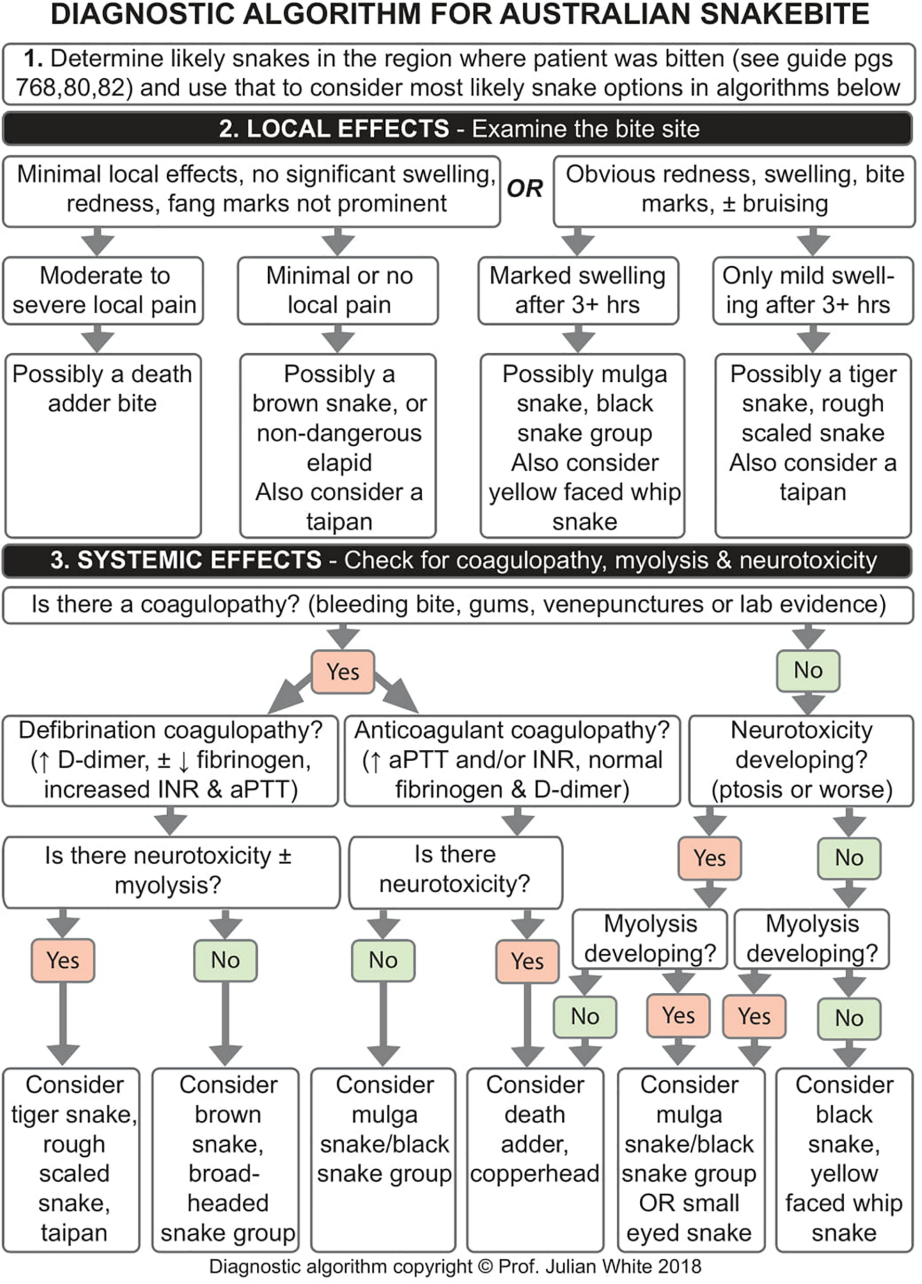
Diagnostic algorithm for snakebite envenoming in South Australia. Algorithm copyright ^©^ Prof. Julian White.

Precise epidemiological data on snakebites in Australia are not available; however, one estimate suggests between 500 and 3,000 snakebites occur annually (118), while another study reported 6,123 hospital admissions due to contact with venomous snakes in a period from August 2001 to May 2013, or an average of about 500 cases per year (119). Snakebite envenoming in Australia is not common, but can be severe with an average of 2.2 deaths annually in the past 15 years with out-of-hospital cardiac arrest being the most common cause (120, 121). Most medically significant snakebites can be attributed to five terrestrial snake groups: Brown snakes (*Pseudonaja* spp.), members of the tiger snake group (*Notechis scutatus, Tropidechis carinatus, Austrelaps* spp., and *Hoplocephalus* spp.), black snakes and mulga snakes (*Pseudechis* spp.), taipans (*Oxyuranus* spp.), and death adders (*Acanthophis* spp.) (18, 109, 121). Some of these snakes can easily be confused by people without experience in identifying snakes. For example, a snakebite victim may report to have been bitten by “a brown snake”, which could belong to any number of species, e.g. the eastern brown snake (*Pseudonaja textilis*) or the king brown snake (*Pseudoechis australis*) (see **Figures 1A, B**), the clinical significance and treatment of which would differ. Diagnostic algorithms can aid clinicians in determining the snake species most likely to have caused the bite, as exemplified in **Figure 2** (109, 116).

Major local effects, such as hemorrhagic blebs and necrosis after snakebites, are rare in Australia and minimal for the brown snakes that cause most cases of snakebite. Nonetheless, some species may cause at least moderate local swelling, and local bruising can uncommonly occur following bites by those species causing defibrination coagulopathy (116). Systemic effects vary depending on species and may include neurotoxic flaccid descending paralysis, systemic myolysis, defibrination coagulopathy, anticoagulant coagulopathy, acute kidney injury (AKI), sudden collapse, cardiac collapse/arrest, anaphylaxis, and microangiopathic hemolytic anemia (MAHA) (109, 116). Death adders, taipans, tiger snakes, and the rough scaled snake commonly cause neurotoxicity; however, tiger snakes, the rough scaled snake, and taipans can also cause myotoxicity. Black snakes and mulga snakes cause myotoxicity and anticoagulant coagulopathy, while defibrination coagulopathy (referred to by some authors as “venom-induced consumption coagulopathy”, “VICC”) is frequent for brown snakes, tiger snakes, rough scaled snake, broad headed snakes (*Hoplocephalus* spp.), and taipans (116). Defibrination coagulopathy can be diagnosed based on an elevated INR of blood clotting and aPTT and grossly elevated D-dimer; the latter may be the first evidence of developing coagulopathy, before any changes in INR of blood clotting and aPTT occur (109). In case of anticoagulant coagulopathy, fibrinogen and degradation products are at normal levels, and aPTT and possibly INR of blood clotting can be prolonged/elevated, whereas defibrination coagulopathy leads to decreased or undetectable levels of fibrinogen and elevated levels of degradation products, both D-dimer and FDP (109, 116). Typically, symptoms of coagulopathy are seen early, sometimes upon arrival to the emergency department, while neurotoxicity and myotoxicity take hours to develop with CK levels peaking between 24-48 hours after the bite (116).

If severe envenoming is diagnosed, diagnostic algorithms in conjunction with the Seqirus (formerly Commonwealth Serum Labs, CSL) Snake Venom Detection Kit (SVDK) can help determine which snake venom immunotype is involved (109). The SVDK is a non-laboratory, rapid, freeze-dried, immunoassay kit, developed for Australian and some Papua New Guinea snake venoms, that uses bite site swabs, or alternatively a urine sample, to detect the venom immunotype. The SVDK is widely distributed and available in Australia, but its usage has declined, in part because of concerns over accuracy and reliability. The reliability of the SVDK is debated due to a high risk of false positives when the SVDK is inappropriately tested on non-envenomed patients, the occurrence of false negatives with envenomed patients (121), and the presence of a hook effect (also known as prozone effect – an effect which describes how the measured analyte concentration can decrease even as the actual concentration increases) (122). The propensity for false positives has proven especially problematic, as the SVDK has frequently been used inappropriately for all suspected snakebites, sometimes as a screening tool – a function that it was not designed for and is not suitable for. One study suggests that false negatives are often a result of operator errors (123), and perhaps for this reason, there is now an annual quality assurance process for all laboratories using the SVDK to minimize the likelihood of operator errors. Antivenom is available at 750 hospitals across Australia, and if an immunotype can be determined *via* the SVDK or otherwise, the appropriate “monovalent” antivenom can be selected as treatment (109). In addition to the five terrestrial “monovalent” antivenoms, a polyvalent antivenom against the five snake groups is also available (121). If the diagnostic algorithms and the SVDK results are in conflict, then either polyvalent antivenom or an appropriate mixture of two “monovalent” antivenoms should be used, but the large volume of antivenom needed, particularly if using polyvalent, represents a potential increased risk of adverse reactions (7, 109). If a clinician is in doubt when handling suspected or confirmed snakebite cases, then advice may be sought *via* the antivenom producer (Seqirus, Melbourne) or through the Clinical Toxinology service (Women’s & Children’s Hospital, Adelaide) (109).

From 2005 to 2015, the median dose of antivenom administered to Australian snakebite victims has decreased from four vials to one vial, with debated implications for treatment (121, 124). Meanwhile, the median time to first antivenom administration has remained unchanged at 4.3 hours (121), despite increasing evidence of a more favorable outcome when antivenom is administered early (125, 126). This lack of change in time to antivenom administration might be because Australia covers a large landmass, with many areas being remote from major health services, making delays in treating snakebites more likely to occur, particularly in remote sites, where antivenom is not stocked and aeromedical retrieval is required. Competing demands on aeromedical retrieval services can exacerbate delays. Additionally, many dangerously venomous snakes in Australia only cause envenoming in a minority of cases. As prophylactive antivenom administration can negatively affect the patient, antivenom should not be administered until it is certain that the patient has been envenomed; this can necessitate further delays in antivenom administration as symptom development is monitored. For these reasons, it seems unlikely that the time to antivenom administration will improve significantly in Australia.

## Snakebite Diagnosis in Asia

The epidemiology of snakebite envenoming differs across Asia as a result of high inter- and intra-species diversity and varying population density of venomous snakes. The impact of snakebite is relatively high in many countries in South- and Southeast Asia, where the overall estimated mortality rate is 1.05 to 5.42 deaths per 100,000 people (4). This includes the Philippines, Thailand, Vietnam, Laos, Cambodia, Malaysia, Myanmar, Nepal, Pakistan, Sri Lanka, and India, where envenomings are predominantly incurred from the following snakes: Cobras (*Naja* spp.), kraits (*Bungarus* spp.), Russell’s vipers (*Daboia* spp.), saw-scaled vipers (*Echis* spp.), Malayan pit viper (*Calloselasma rhodostoma*), hump-nosed pit vipers (*Hypnale* spp.), and green pit vipers (*Trimeresurus* spp.) (114, 127–129). In Japan, Korea, Hong Kong, Taiwan, and Indonesia, most envenomings are caused by pit vipers (subfamily: *Crotalinae*), which might be associated with lower mortality rates (130–134). Data on the epidemiology of snakebite in Central-, West-, and North Asia, including Russia and the Middle East, are limited, but estimates suggest that the rates are low compared to the subtropical and tropical regions of South- and Southeast Asia (4). Similarly, data on the epidemiology in China are limited, with one study suggesting that mortality rates in East Asia, including China, range from 0.033 to 0.347 per 100,000 people (4).

The majority of physicians in South- and Southeast Asia rely on the circumstances of the bite and clinical manifestations to diagnose the victim (135). Syndromic diagnostic tools and algorithms are available for Southeast Asia in general (SEARO guide (136)) and for some countries in particular (e.g. Myanmar (9, 137, 138)). Similar to Australia, a thorough patient history can be helpful in identifying the type of snake involved in the accident. E.g. if a victim has been bitten in a house during the night and has developed paralysis, the culprit is more likely to be a krait (*Bungarus* spp.), while a bite sustained from a venomous snake in a tree might suggest a green pit viper (*Trimeresurus* spp.) (135, 139, 140). Systemic signs of envenoming can also be helpful in clinical practice, as the venom of most species in South- and Southeast Asia are mainly toxic to either neuromuscular or hemostatic systems. Neurotoxicity is often related to bites by cobras, king cobras, and kraits, while hemotoxicity usually indicates envenoming by a true viper (subfamily: *Viperinae*) or pit viper, although in rare cases it may indicate envenoming by a colubrid, such as a red-necked keelback (*Rhabdophis subminiatus*) or a tiger keelback (*R. tigrinus*) (139). It can sometimes be difficult to differentiate between neurotoxic envenoming by cobras and kraits based on clinical signs. However, krait bites are often associated with delayed onset and prolonged total period of paralysis, while cobra bites are often associated with significant local evidence of envenoming (136, 141). Behavioral differences of the snakes might further elucidate the matter, as krait bites primarily occur at night, while cobra bites are much more likely to occur during the day (135, 140). When documenting the clinical manifestations of envenoming, some clinicians use a standardized questionnaire based on national snakebite management guidelines to support the diagnostic process (142). A systematic syndromic approach combined with a scoring system based on clinical manifestations has been proposed to assist clinicians in identifying the offending snake species (143), but sufficient data on envenoming profiles to create such systems are lacking for many species throughout South- and Southeast Asia (137, 143).

India has more snakebites and snakebite-related deaths per year than any other country in the world (135, 144). It is home to 52 venomous snake species, out of which the Russell’s viper (*D. russelii*), the common krait (*B. Caeruleus*), the Indian cobra (*N. naja*), and the saw-scaled viper (*E. Carinatus*), known as the “Big Four”, are considered the most medically important. Both polyvalent and monovalent antivenoms are available in India, but they do not cover all venomous species. Furthermore, there is a need for a standardized quality control process for manufacturing of snakebite antivenoms to ensure that they are safe and effective (145). National guidelines for management of snakebites in India do exist and some states have developed their own protocols. However, in many cases, these protocols are not followed strictly, leading to misdiagnosis and inappropriate management (146). As an example, hump-nosed pit vipers (*H. hypnale*) are often misidentified as saw-scaled vipers (*E. carinatus*), resulting in administration of ineffective antivenom (141). Furthermore, some doctors and hospitals are unwilling to manage snakebite victims, causing potentially critical treatment delays (147). A diagnostic tool for identification of the offending snake species combined with a coordinated approach to ensure that healthcare workers across India have adequate knowledge, skills, and confidence to manage snakebite patients could potentially reduce this problem.

Snakebite envenoming remains an important health issue in many regions of Asia, especially throughout South- and Southeast Asia, where incidence and mortality rates are among the highest in the world. The high species diversity complicates clinical management, although this problem is alleviated somewhat by the widespread use of polyvalent antivenoms. While these polyvalent antivenoms are convenient for physicians, they may arguably be disadvantageous for overall patient outcomes, as they can be a disincentive for quality epidemiologic and clinical envenoming studies. Uncertainty about the offending snake species may result in masking of “new” envenoming syndromes, thereby hampering the inclusion of new species into antivenom immunization protocols. The continuing absence of *Hypnale* spp. from the immunizing mix for Indian polyvalent antivenoms (148–150) can be mentioned as an example of this.

## Snakebite Diagnosis in the United States and Canada

In the United States (US) and Canada, around 6,500 people suffer from snakebites annually, resulting in 5-6 deaths (5, 151, 152). The US has about 26 indigenous venomous snake species, where rattlesnakes (*Crotalus* spp.), moccasins (*Agkistrodon* spp.), and pygmy rattlesnakes (*Sistrurus* spp.), all of which belong to the pit viper (*Crotalinae)* subfamily, are the main genera implicated in snakebites. Coral snakes (*Micrurus* spp.) are also present in a limited southern distribution but do not account for many bites (153), with an estimated 70-80 annual cases reported to the American Association of Poison Control Centers. In Canada, rattlesnakes are the only medically relevant snake species, and with a very limited distribution, the risk of snakebite is relatively small (154).

Pit vipers are the most prolific group of snakes involved in snakebite accidents in the US and Canada; therefore, when managing a snakebite patient, it is important to keep in mind that less than 10 percent of pit viper bites are dry bites (155, 156). Pit viper venom typically contains hemotoxins causing direct or indirect lysis of fibrinogen, thrombocytopenia, and vascular endothelial damage (157, 158), thereby emphasizing the importance of carefully monitoring the patient’s blood coagulation profile through laboratory tests. Furthermore, the presence of Mojave toxin in Mojave rattlesnake (*C. scutulatus*) and southern pacific rattlesnake (*C. helleri*) venoms causes potentially severe systemic neurotoxicity, including cranial neuropathy and flaccid paralysis. Severe neurotoxic clinical manifestations, when present, are a relevant diagnostic indicator (159–161). It is recommended to perform laboratory tests of the patient every 6-8 hours and twice prior to discharging the patient in order to follow the progression of the envenoming (110).

Coral snake envenoming generally causes only mild local effects, while the systemic manifestations can include euphoria, lethargy, nausea, vomiting, excessive salivation, ptosis, dyspnea, convulsions, abnormal reflexes, and motor weakness or paralysis leading to respiratory paralysis, which is lethal in absence of clinical intervention (162–165). In case of a coral snake envenoming, serum creatine kinase activity may rise, and myoglobin may be detected in the urine (164, 166), but coagulopathy is not a feature (165–168). The observation time in the hospital depends on the severity of the envenoming, the age of patient, and the location of the bite wound, ranging from at least 8 hours to 12-24 hours for mild envenomings, where repeated laboratory evaluations are advised (110). The marked visual appearance and clinical presentation of coral snake envenomings in the US make coral snake envenomings easy to distinguish from pit viper envenomings. The genus-specific antivenom, Pfizer Antivenin, has been available for the treatment of coral snake envenoming but is currently in very short supply, resulting in rationing (162, 169), though the recent recommencement of production should alleviate this shortage.

Like several other countries, the US also has a treatment algorithm: The unified treatment algorithm, published in 2011, with the purpose of streamlining the management and diagnosis of snakebites in the US (110). However, since the algorithm was published, a new antivenom has become available, and the algorithm has not yet been updated accordingly. As Canada does not have indigenous snake species that are different from those in the US, it is likely that this algorithm is applicable to assess snakebite cases in Canada as well.

Clinicians in the US will often factor in information provided by the victim or bystanders about the identity of the snake. A study comparing the snake identifications of expert herpetologists with those of snakebite victims, witnesses, and healthcare providers in southern parts of the US found that 40% of the specimens identified as copperheads (*A. contortrix*) were actually cottonmouths (*A. piscivorous*), with juvenile snakes being particularly difficult to identify, leading to confusion (170). While other species were less frequently confused, it might be problematic that (possibly erroneous) snake identifications are used by poison control centers when recommending treatment (170). Although pit viper bites in the US are treated with polyvalent antivenom (CroFab or AnaVip) when required, misidentification of pit vipers might still negatively impact treatment. For example, AnaVip, which is based on equine F(ab’)_2_ antibodies, has proven more efficient in treatment of late onset and recurrent coagulopathy than CroFab, which is based on ovine Fab antibodies (171). This difference in efficacy versus coagulopathies might be related to the different half-lives of Fab and F(ab’)_2_ antivenoms (171). Both CroFab and AnaVip are recommended for treatment of rattlesnake envenoming in North America, but AnaVip has not received FDA-approval for treatment of bites by cottonmouths and copperheads. Conversely, CroFab works well for treatment of copperhead (*A. contortrix)* bites, by decreasing limb disability subsequent to bites (172) and being associated with fewer patients using opioids to treat pain related to the envenoming (173). It has additionally been demonstrated that early administration of CroFab for copperhead bites results in faster limb recovery than does late administration (174). Thus, in cases of copperhead envenomings, it might be especially beneficial to rapidly identify the culprit species so the optimal polyvalent antivenom can be administered early on.

## Snakebite Diagnosis in Latin America

In Latin America and the Caribbean islands, 80,000-129,000 snakebite envenomings occur each year, leading to an estimated 540-2,300 deaths (4). Throughout the Latin American countries, bites from lanceheads (*Bothrops* spp.) are the most prevalent. Rattlesnakes (*Crotalus* spp.), bushmasters (*Lachesis* spp.), and Coral snakes (*Micrurus* spp.) are also present, but especially the latter two are far less common causes of snakebites (175, 176). In Central America, snakebites are also caused by moccasins (*Agkistrodon* spp.), jumping pit vipers (*Atropoides* spp.), palm pit vipers (*Bothriechis* spp.), montane pit vipers (*Cerrophidion* spp.), and hog-nosed pit vipers (*Porthidium* spp.). The venoms of pit vipers indigenous to Central America can be treated with polyvalent antivenom (175). The clinical utilization of polyvalent antivenom makes diagnosis at a species or even genus level less important, as noted earlier for Asia and the US and Canada. However, it is important to determine which family (viper, elapid, or other) the perpetrating snake species belongs to, whether an envenoming has taken place, and the severity of the envenoming (175). For South American countries, both polyvalent and genus-specific antivenoms are available (111, 176–179).

Several Latin American countries have protocols for diagnosis and treatment of snakebite envenoming, describing the use of the syndromic approach and the laboratory investigations mentioned in **Table 2** (111, 175, 176, 178–180). Several of these protocols mention coagulation time as a commonly investigated parameter for early detection of a pit viper envenoming (111, 175, 176, 178–180). Often in pit viper envenomings, the 20WBCT is positive (no clot at 20 minutes), while for elapids it remains negative (normal clot at 20 mins) (165–168). In Mexico, where rattlesnakes are the predominant genus, the Lee-White clotting time (LWCT) is utilized to determine the presence of coagulation disorders, which can in turn give an indication of the urgency of commencing treatment (180). LWCT is fundamentally similar to the 20WBCT described earlier, with the only difference being that the LWCT is observed once per minute after an initial incubation time of five minutes (181). The effectiveness of LWCT was assessed in Brazil for its sensitivity toward detecting coagulopathy in lancehead envenomings and was considered a valuable tool in evaluating the need for antivenom therapy (181).

Pit viper envenomings may cause both local and systemic effects, but there are two distinct patterns. Most Central and South American pit vipers cause moderate to severe local effects and coagulopathy, often with hemorrhagic features. The exception is rattlesnakes, which are more likely to cause major systemic effects including neurotoxicity, rhabdomyolysis, and coagulopathy, while only causing mild local effects. Local effects following pit viper bite, with the exception of rattlesnakes, may include edema, severe local pain, swelling, local hemorrhage, inflammatory erythema, lymphangitis, bleeding from the bite wound, blistering, ecchymosis, tissue necrosis, and secondary infections (7, 111, 175, 176, 178–180, 182–185). Systemic effects may include early syncope, confusion, transient loss of vision or darkening of vision, hypotension, shock, renal damage, cardiac tachyarrhythmia or bradyarrhythmia, coagulopathy, and systemic hemorrhage (7, 111, 175, 176, 178–180, 182, 183, 185). With the knowledge of which snakes induce which clinical manifestations, the syndromic approach works well and is widely used. However, the approach requires thorough knowledge of the different venomous snakes (7, 186) and relies on the presence of polyvalent antivenoms targeting the venoms of one or multiple genera.

The similarity between the local effects of lancehead species and bushmaster species makes differentiating the two a challenging task, which can be further complicated by the fact that, in some regions, both genera are known locally as “surucucu” (see **Figures 1C, D**). However, the vagomimetic effects, sometimes induced by bushmaster venom on the gastrointestinal system, may cause diarrhoea, thus indicating the most likely genus of the culprit snake. Although this can be a strong indicator, the lack of such effects does not exclude the presence of a lachetic envenoming (151, 176), nor do their existence confirm it.

Unlike bites from lanceheads and bushmasters, many rattlesnake bites are more easily recognized by the neurotoxic effects that they can inflict. South American rattlesnakes (*C. durissus*) generally do not cause severe local manifestations but instead induce neurotoxicity resulting in neuromuscular paralysis (183, 184, 187), caused by neurotoxic crotamines and crotoxins present in the venoms. Envenomings by South American rattlesnakes often lead to mild to severe neurotoxic manifestations in the patient, which are clinical hallmarks that may guide the physician toward a correct diagnostic assessment (111, 159–161, 176, 178, 180). However, it has been reported that envenomings by juvenile South American rattlesnakes can result in coagulopathy as the main systemic manifestation, instead of neurotoxicity, which may lead to misdiagnosis and administration of wrong antivenom (188).

Coral snake envenomings are associated with very different clinical manifestations, such as local paresthesias, vomiting, muscle paralysis including paralysis of respiratory muscles, ptosis, ophthalmoplegia, diplopia, and late manifestations including secondary renal damage and respiratory failure (111, 168, 175, 176, 178–180, 183, 184). Although clinical manifestations may overlap with those of some rattlesnakes, the recognizable color schemes of coral snakes make a strong case for coral snake envenoming. Coral snakes found in most of the Pan-American countries are visually very distinct from pit vipers. However, nonvenomous snake species mimicking the venomous coral snakes exist (e.g. milk snakes: *Lampropeltis triangulum*). These are difficult to distinguish by a non-professional, but guidelines based on the color scheme of the snakes can be found that aid in the differentiation (162, 175, 189).

Snake biodiversity varies significantly throughout Latin America, from Argentina inhabited by three medically important snake genera (*Bothrops*, *Crotalus*, and *Micrurus*) to the plethora of medically important species found in Mexico and the Central American countries (190). This shift in indigenous snake species greatly impacts the diagnostic approach, where the severe local effects of lancehead envenomings become a specific indicator in Argentina (111), but is easily confused for a lachetic envenoming in Brazil (176). Polyvalent antivenoms alleviate the dependence on successful determination of the species of the culprit snake by simply requiring successful assessment of the snake family involved. However, as discussed previously, there may be disadvantages to being restricted to polyvalent antivenoms, and different polyvalent antivenoms may perform differently in a given clinical case.

## Snakebite Diagnosis in Africa

The extent of the snakebite problem in Africa is difficult to assess due to the scarcity of epidemiological data (182). However, of all the African regions affected, snakebite is most commonly observed in sub-Saharan Africa, where an estimated 90,000-420,000 envenomings occur annually, resulting in 3,000-32,000 deaths (5). In comparison, an estimated number of 3,000-80,000 bites occur in North Africa and the Middle East combined, leading to 4,000-8,000 deaths annually (5). To the best of our knowledge, a combined mortality rate for all of Africa has not been recorded, but it has been estimated that some of the populations most vulnerable to snakebite worldwide are found in Africa (191). The snakes that are responsible for the majority of bites and are associated with serious or life-threatening envenomings are saw-scaled vipers (*Echis* spp.), large African adders or vipers (*Bitis* spp.), spitting or cytotoxic cobras (*Naja* spp.), neurotoxic cobras (*Naja* spp.), and mambas (*Dendroaspis* spp.) (192). In addition to the potency of the snake venoms themselves, factors potentially contributing to the high mortality rate may include scarcity of antivenoms (partially due to the high cost of antivenoms relative to personal income levels), low quality, inappropriate, or counterfeit antivenoms, suboptimal health services, difficulties with quick access to health centers, and insufficient training in clinical snakebite management, including a lack of diagnostic training and/or tests (2, 193–196).

In many African cases, appropriate clinical management of snakebite patients requires identification of the distinctive clinical syndrome based on epidemiological, clinical, and laboratory data (e.g. 20WBCT), and consequently the syndromic approach is often recommended (15). Researchers and clinicians have sought to objectively quantify the severity of snakebite envenoming to minimize confusion due to the ambiguity of the definitions offered by current guidelines (197). In Southern Africa, five main clinical syndromes of snakebite envenoming are recognized and often these guide diagnosis: Local pain and progressive swelling (cytotoxicity), progressive paralysis (neurotoxicity), incoagulable blood (hemotoxicity), moderate to marked local swelling (associated with otherwise neurotoxic bites), and mild to moderate swelling, with negligible or absent systemic effects (neurotoxicity and cytotoxicity) (15). However, with the syndromic approach, it is possible to misidentify snake species due to the similarity between symptoms that develop following envenoming from different types of snakes. For instance, mixed hemorrhagic and cytotoxic symptoms develop following envenoming caused by saw-scaled vipers, puff adders (*Bitis arietans*), and horned desert vipers (*Cerastes cerastes*) (see **Figures 1E, F**) (192).

Several polyvalent and a few monovalent antivenoms have been marketed for the treatment of envenomings caused by African snake species, but the antivenoms are not necessarily equally appropriate for the treatment of bites from a given genus or species, in spite of being marketed as such (198–201). The antivenoms are also not evenly distributed throughout the continent, and some areas have been plagued by antivenom shortages (2, 193, 196, 202). It might therefore be expected that the disparity in the availability and types of antivenoms in Africa is reflected by a variability in the demands for diagnosis. However, to be diagnosed or treated, the patient must make their way to either a health center or a properly trained clinician, which may often be difficult or result in long delays. A study published in 2015 estimated that about 29% of the population in Africa are geographically marginalized from emergency medical care and live more than two hours from the nearest public hospital (203). The same study found that only 16 of 48 countries have more than 80% of their population living within two hours’ travel time of emergency hospital care (203). Thus, it is no surprise that many snakebite victims in rural communities resort to seeking out traditional healers, rather than trained physicians (195). This trend is also observed outside of Africa, when looking at other rural parts of the world that are heavily burdened by snakebite (204–209). This delay in receiving proper medical care will, in most cases, worsen the symptoms and thus increase the likelihood of a poorer clinical outcome (206, 210).

For diagnosis, management, and treatment of snakebite victims in Africa to improve, it will be essential to address the knowledge gap between the health institutions, rural communities, and their local traditional healers (206). One strategy to approach this is *via* outreach and education programs promoting snakebite prevention and first aid (211). Such programs could even include traditional healers in an attempt to utilize their status as authority figures (211), rather than attempting to fight strongly-held community cultural beliefs. Also, increased availability of mobile phones with inbuilt cameras could facilitate the involvement of (distantly located) expert herpetologists in snake species identification without the need to capture or kill the snake (206, 212).

## Snakebite Diagnosis in Europe

Snakebite incidents are a relatively rare occurrence in Europe with an incidence rate of 1.06 bites per 100,000 people and about 4 deaths annually (213). Contrary to what some believe, snakebites from species indigenous to Europe can cause severe envenoming and require immediate medical attention. All significantly venomous snakes in Europe belong to the *Viperinae* subfamily, with the common European adder (*Vipera berus*), European asps (*V. aspis*), and common sand adder (*V. ammodytes*) being responsible for the largest proportion of severe envenomings (213, 214).

Many areas of Europe are inhabited by only one species of venomous snake, especially in Northern and Central Europe (213, 214). If diagnosis is necessary in areas with more than one species, it is usually based on witness statements, a picture of the culprit snake, or the snake itself brought by the victim (215–217). In severe cases, the presence of neurotoxicity can be an indication that the envenoming was caused by either a common sand adder or a European asp, as these two species are the most common causes of neurotoxicity due to envenoming by indigenous European snakes. Additionally, because these species have disjunct distributions, neurotoxicity can help pinpoint exactly which species caused the bite (213, 218). However, the absence of neurotoxicity does not exclude European asp bites, as most subpopulations do not possess neurotoxic venom (219). Neurotoxic clinical features have also occasionally been reported after envenoming by the common European adder, but this has been limited to a few geographical areas in Eastern Europe and has mostly been caused by the subspecies known as the Bosnian viper (*V. berus bosniensis*) (216, 217, 220). For this reason, in most of Europe, elaborate laboratory tests for diagnosis of the culprit snake species is a low priority. However, laboratory tests are used to assess the severity of envenoming, and thereby the need for antivenom (214, 221). Clinical manifestations monitored include hypotension, neurologic or gastrointestinal symptoms, edema, and leukocytosis. A full overview of clinical manifestations is given elsewhere (222).

Snakes inject a variable amount of venom and dry bites can occur (223–226). Victims are normally admitted for observation for 24 hours to monitor possible symptom progression (225, 227). Despite the impracticality of using clinical signs for diagnosing the species involved in most European snakebites, the severity of the symptoms and signs can be used to determine the need of antivenom administration in moderate to severe envenomings. A grading system for assessing the severity of an envenoming has been proposed based on data on the appearance of clinical manifestations from common European adder and European asp cases and has been used as a guideline in research and in certain clinical settings (53, 221, 225, 228).

Despite the close phylogenetic relationship between *Vipera* spp., inter- and intraspecific venom variability might occur, both with regard to the toxins present and their individual abundances, which, in turn, may affect antivenom efficacy (213, 229–231). However, available monospecific antivenoms may still show cross-reactivity between venoms, and studies have shown that antivenom raised against venom from one species can, in some cases, have clinical efficacy against venoms from other vipers indigenous to Europe (214, 215, 232).

Bites by exotic snakes are not as prevalent as those by indigenous species. However, they are still the cause of a few severe bites around Europe, mostly affecting amateur snake keepers (233–235). In these cases, rapid identification of the responsible species is important as it can help predict clinical manifestations and aid symptomatic treatment. As the snake is not endemic to the country, clinicians will usually rely on statements from witnesses for identification, and required antivenom should be sourced as soon as possible as it might not be stocked in the given country (236) (exotic antivenom banks exist in a few countries, e.g. the Netherlands and Germany).

## Potential Benefits of Novel Snakebite Diagnostics

Studies find that early treatment of Australian and North American snakebite victims is linked to faster recovery and shorter time to hospital discharge (126, 174). In a similar vein of inquiry, it was established that delays in treatment increase the risk of acute kidney injury in snakebite victims in Myanmar and the risk of acute renal failure and the overall severity of envenoming in snakebite victims in Brazil (237–239). One of the studies also found that patients who developed acute renal failure required more antivenom and were hospitalized for a longer period of time than those who did not (238). These studies point to the unsurprising conclusion that delays in treatment often negatively impact patient outcome, which in turn can result in prolonged hospitalization time and increased resource consumption at the treatment facility. It thus seems plausible that improved diagnostics might enable rapid diagnosis and thereby facilitate early and correct treatment, as well as improved patient outcomes. This is backed by a recent study of 742 snakebite patients in Sri Lanka, which argues that delays in antivenom administration reflect an absence of diagnostics for early detection of envenoming, and that such diagnostics are required for improved, early treatment with antivenom (240). Novel diagnostics will likely have the greatest impact in areas where transportation to the treatment facility and antivenom availability are not limiting factors, areas with many different indigenous snake species that are visually difficult to discern, areas where monovalent antivenoms are available, and areas with medical or paramedical personnel with limited training in clinical management of snakebite envenoming.

In addition to their utility in supporting clinicians in diagnosing snakebite patients and choosing the correct antivenom on a case-by-case basis, novel snakebite diagnostics could also be of interest on a grander scale. They could enable epidemiologists to map patterns of snakebite incidence. In turn, knowing which snake species are responsible for the majority of bites in an area can help authorities manage their resources, when deciding which antivenoms to procure in which quantities, and where to deploy them within a healthcare system (241). Improved diagnostics might also inform the design of novel antivenoms, and they could become indispensable tools for clinical trials of future generations of antivenoms, and later (if adopted as companion diagnostics) in clinical snakebite management. Based on the potential use cases and benefits listed above, it is perhaps hardly surprising that researchers and physicians have indicated the need for improved diagnosis of snakebite victims for decades (7, 83, 240, 242–248).

## Snakebite Diagnostics Reported in the Literature

Several diagnostic assays have been developed to meet the demand for improved diagnosis of snakebite victims. The diagnostics rely on techniques varying from immunoassays (typically ELISAs), over enzymatic activity assays, to forensic genetic methods (see **Table 1**). These studies demonstrate that snakebite envenoming can be diagnosed using various technologies, and they showcase the development of snakebite diagnostics throughout the past six decades. As evident from **Table 1**, there has been a gradual shift in the preferred methodologies from radioimmunoassays and agglutination tests over the ever-popular ELISA format, toward an increased focus on LFAs and more diverse non-immunological methods. As a reflection of this technological progression, the experimental diagnostics reported in literature have become faster over time, although interestingly, their limits of detection do not seem to have improved significantly. One hypothesis explaining this could be that, while faster immunoassays have been developed, the antibodies at the core of these assays are essentially unchanged, with most still being derived from horses, rodents, and lagomorphs (see **Table 1**).

Many of the earliest reported diagnostic tests for snakebites were developed for first-world countries, with Australia being prominently featured (see **Table 1**). However, this trend has changed, and snakebite diagnostics have now been developed for countries all over the world. As an example, in Brazil, an ELISA-based diagnostic tool has been utilized experimentally to aid differential diagnosis on a genus level (176). Similar assays have been developed that make it possible to evaluate the effectiveness of the antivenom administered to neutralize the venom (176). More recent examples of innovation within snakebite diagnostics in Brazil include an impedimetric immunosensor based on electrochemical impedance spectroscopy (86) and the use of infrared thermography (94). Meanwhile in Asia, Hung et al. developed a sandwich-type enzyme-linked immunosorbent assay (ELISA) capable of detecting Taiwan cobra (*N. atra*) venom in biological samples with a detection limit of 1 ng/mL (72). The same group later developed an immunochromatographic strip to detect Taiwan cobra venom in patient serum in only 20 minutes (82), while a different group similarly developed an ELISA and an immunochromatographic strip for diagnosis of snake species in Taiwan (85). A number of other molecular diagnostic PCR-based tests for stratifying venom from Asian snake species have also been reported. However, these tests typically take at least 3-4 hours to complete and have lower specificity compared to immunoassays (90–92, 249). Generally, issues with cross-reactivity of the tests toward several species remains a problem for rapid diagnosis of snakebite envenoming, and many reported rapid diagnostic methods are not reliable enough for clinical use and can only be used for research purposes (30, 40, 71, 73, 76, 77).

Although the studies referenced above clearly demonstrate that snakebite diagnostics can be developed for the stratification of many snake species and using many methods, to the best of our knowledge, the SVDK is the only snakebite diagnostic to have been adopted in the clinical setting. The success of the SVDK in Australia may reflect the preference there for using monovalent antivenoms, unlike many other countries, which rely on polyvalent antivenoms. This reliance could create a barrier for adoption of venom detection tests. Generally speaking, the reason for the low adoption rate for novel diagnostic assays is not entirely clear, but a variety of explanations of both technical, financial, and implementational nature are likely to be part of the underlying cause (250). The antivenom market is notoriously financially unstable in many regions (196), and if this is any indication, it leaves little financial incentive for marketing snakebite diagnostics. To exacerbate the problem, snakebite diagnostics are perhaps above all else needed by clinicians in remote healthcare facilities with no training in clinical snakebite management. A lack of education in snakebite management among the users of future diagnostics might complicate the implementation of the diagnostics. Even if these and other financial and implementational challenges can be surmounted, a number of technical pitfalls still exist that one needs to be aware of. Below follows a discussion of some of these pitfalls and design considerations that developers of snakebite diagnostics should take into account to avoid them.

## Design Considerations for Snakebite Diagnostics

When developing a diagnostic for a Neglected Tropical Disease, one of the most important factors to consider is affordability. The association between snakebite envenoming and poverty greatly affects the availability of treatment (2, 7, 11, 12, 251, 252), and this link between affordability and availability is likely to also exist for diagnostics. Affordability may place restrictions on the types of equipment required to use the diagnostic, especially at small, remote treatment facilities, where access to electricity can be unreliable, and for point-of-care testing. Point-of-care testing additionally requires greater user-friendliness, as the person carrying out the test may have received only limited or no training in its use. For these reasons, a PCR with a low limit of detection and a requirement for specialized laboratory equipment and knowhow, such as that developed by Supikamolseni et al. (91), and a user-friendly lateral flow assay with a higher limit of detection, such as that developed by Liu et al. (85), may be differentially suited for use at centralized treatment facilities and point-of-care settings, respectively. However, with the implementation of different types of PCR [see e.g (253–255)], it will likely be possible to make fast, user-friendly, PCR-based diagnostics for point-of-care testing in the future.

The sample matrix and sampling method should also be considered and as far as possible be adapted to the intended use case. In a healthcare facility, it may be convenient to use blood samples for diagnostics, as it is a standard procedure to take blood samples from snakebite patients for use in the existing laboratory diagnosis (192, 256). However, in point-of-care use cases, wound swabs and exudates may be more readily available. While the sampling method affects user-friendliness, the sample matrix may affect the technical specifications of the diagnostic, as different sample types are likely to contain different concentrations of the analyte at different time points, as well as different concentrations of interfering substances (i.e. substances that alter the detected concentration of the analyte). For example, blood samples have notoriously complex compositions compared to e.g. urine samples, and this increases the risk of blood samples containing interferants. Conversely, the collection of blood samples at healthcare facilities is a highly standardized procedure, unlike the collection of wound swab samples, which may additionally be affected by subjection of the bite wound to inappropriate first aid methods or other forms of tampering. Being collected from the surface of the body, wound swab samples may not be representative of the amount of venom actually delivered into the body of a victim, although they may still provide valuable information about the type of snake involved. As demonstrated in **Table 1**, diagnostics have been developed and tested on various different sample matrices, including blood (and as derivatives hereof: Plasma and serum), urine, tissue samples, wound exudate, and wound swabs. Preferences for the sample matrix vary, with some researchers placing more emphasis on standardization and how well the venom content in the sample type reflects the venom content at the active sites in the body, while others emphasize user-friendliness and a low risk of interference from other sample components. Perhaps to account for the advantages and disadvantages of the various sample types, some assays, e.g. the SVDK from Australia, function with multiple different sample matrices (257).

An additional factor to consider is the time required to use the diagnostic. Because snakebite envenoming is acute in nature, with some toxins exerting their effects within minutes, it would likely be beneficial for a diagnostic device intended for clinical use to function on a timescale of minutes rather than hours. Conversely, for forensic and purely epidemiological studies, rapid assay time may not be a requirement. Therefore, time-consuming diagnostics, such as ELISAs with overnight incubations, may be as well-suited for retrospective diagnosis as more rapid assays. Furthermore, it is advantageous for the diagnostic tool to be stable over a wide range of temperatures and environmental conditions, as the provision of cold-storage may be problematic in some areas of the world (16, 241).

Important technical parameters, which are not specific to snakebite, also need to be considered, including specificity, sensitivity, and positive predictive value. Low specificity (i.e. the number of true negatives divided by the total number of individuals not suffering from a condition) leads to false positives, as demonstrated in a study by Ho et al. (40), where the researchers set up an ELISA to study snakebites in rural Thailand. Here, non-specific reactions of ELISA reagents led to a false positive rate of up to 75% (40). A study by Isbister et al. demonstrated how low sensitivity (i.e. the number of true positives divided by the total number of individuals with the condition) of the 20WBCT for Russell’s viper envenoming led to a high rate of false negatives, which in some cases resulted in delayed antivenom administration (258). If the sensitivity, specificity, and disease prevalence are known, they can be used to calculate the positive predictive value, using the formula:

$$Positive\;predictive\;value=\;\frac{sensitivity\;\cdot \;prevalence}{sensitivity\;\cdot \;prevalence\;+\;(1-\;specificity)\cdot (1-prevalence)}$$

The positive predictive value is an indication of how likely patients with positive test results are to truly suffer from a condition (e.g. snakebite envenoming sustained by a cobra). Unfortunately, although Ho et al. argued the importance of reporting these measurements of assay performance already in 1986 (40), very few studies involving snakebite diagnostics contain these values, and some diagnostics are not even tested on patient samples (see **Table 1**). The absence of positive predictive values for snakebite diagnostics in the literature may be a reflection of the lack of available data on disease prevalence. As more epidemiological data hopefully becomes available, it may become easier to evaluate the potential of novel diagnostics by using the positive predictive value as a performance measurement.

Additional technical parameters of importance include limit of detection (LoD), quantitativity, and limit of quantitation (LoQ). In the literature, snakebite diagnostics have been reported with limits of detection (i.e. the lowest concentration of a substance that can be distinguished from the absence of the substance) ranging from 0.1 pg/mL to 0.3 mg/mL (see **Table 1**). The limit of detection required depends on the pharmaco-/toxicokinetics of the analyte. For example, if the analyte has a short half-life and a high volume of distribution, the limit of detection in a blood sample will need to be much lower than for an analyte with a long half-life and low volume of distribution. The influence of analyte kinetics is also relevant when discussing quantitative diagnostics. Quantitative diagnostics are interesting, because they can provide information not only about the presence of an analyte but also about its abundance. For quantitative assays, in addition to establishing the limit of quantitation (the lowest amount of analyte that can be quantitatively measured with a certain precision and accuracy), it may also be important to establish threshold values. For example, if the analyte measured is a biomarker of kidney injury, which is also found in low amounts in healthy individuals, it is important to determine a threshold value to distinguish patients with normal amounts of biomarker from patients with abnormal amounts. If the analyte is a snake venom toxin, it might be relevant to determine several thresholds corresponding to commonly used categorizations of “mild”, “moderate”, and “severe” envenomings, which correspond to different treatment strategies. Quantitative diagnostics could potentially also be used to evaluate the effectiveness with which antivenoms sequester toxins, by monitoring unbound toxins in blood samples from patients. This could make quantitative diagnostics useful tools for antivenom performance evaluation and monitoring of patients’ disease progression/envenoming grade alike. A low level of free toxins might mislead non-toxinologist clinicians into believing the patient can be discharged, but due to a depot effect and a mismatch between the toxicokinetics of the venom and the pharmacokinetics of the antivenom, symptoms can recur (259). Potentially, if patients are kept under observation, quantitative diagnostics will enable clinicians to detect an increase in free toxin levels, before symptoms recur, and prepare accordingly. However, the Achilles’ heel of diagnostics relying on toxin detection may be the underlying assumption that the toxin concentration is measurable in a readily available sample, and that this concentration is always reflective of the toxin concentration at the site where the toxin exerts its effects. While some studies have found correlations between venom antigen concentration in patient samples and certain clinical manifestations of envenoming, others have found the opposite (52, 53, 69, 260–262). This dichotomy underlines the complexity of the relationship between toxin concentration and distribution over time (263). The matter is complicated further for both toxin and non-toxin analytes, if the influence of preexisting morbidities on the analyte’s kinetics is factored in. For instance, diseases such as chronic kidney disease may alter the clearance or even the baseline concentration of an analyte (if the analyte is a naturally occurring biomarker, see (264) for examples). The disparity between measured analyte concentration and signs of envenoming might be alleviated by using samples of the affected tissues instead of more distant tissues, e.g. by using a muscle biopsy instead of using a wound swab, if one is trying to assess myotoxicity. However, for clinical (as opposed to forensic) samples, this strategy could be highly problematic, as the risks involved for the envenomed patient may outweigh the potential benefits. If, in spite of these challenges, any meaningful thresholds can be established for quantitative diagnostics, it will be important that assay precision and accuracy are high in the surrounding range. It should also be determined in which range there is a linear correlation between actual and measured analyte concentration, and whether the assay is affected by the hook effect.

As evidenced by **Table 1**, many snakebite diagnostics have been developed using immunological methods, often using equine, leporine, or murine IgGs. For diagnostic immunoassays, it may be worth considering the format and origin of the antibodies used. While format and origin are key decisions that greatly affect antivenom utility (177), these antibody properties may be somewhat less influential in diagnostics, because the diagnostic antibodies are not injected, thus rendering their pharmacokinetics and pharmacodynamics irrelevant. However, it is still possible that endogenous factors in a patient sample (e.g. anti-idiotypic antibodies, other heterophilic antibodies, or factors in a blood sample) can react with heterologous antibodies on a diagnostic test, thereby causing a high background signal, which needs to be accounted for (40). In addition to establishing standard curves and subtracting such background signals, less laborious options are available. For example, it might be possible to use a different sample type that does not contain the problematic factors or to filter the problematic factors out of the originally selected sample type, especially if their sizes are very different from that of the analyte [see e.g (265, 266)]. Alternatively, an excess of unspecific antibody could be added to outcompete the diagnostic antibodies for unspecific binding to the interfering factors. The antibody format should also be considered, as different antibody formats have different avidities and different options for chemical modifications, such as linkage to dyes and tags or attachment to surfaces or larger particles (267, 268).

It is also important to consider which information is of most use for treatment. Several parameters exist that, if measured, could provide useful information to the treating physician. For example, measurement of biomarkers for the development of clinical manifestations is already used to help physicians identify and predict pathologies such as coagulopathies, rhabdomyolysis, and acute kidney injury (7, 109, 256). Detection of snake venom toxins and/or identification of snake species is also of interest, as knowing the snake species or type of venom injected into the victim can aid in deciding which antivenom (if any) is appropriate, as well as it might help predict later clinical manifestations. It may be relevant to distinguish which snakes are of greatest interest to discern from an epidemiological and a clinical viewpoint, respectively. For example, snake stratification at a species level may be very valuable in epidemiological studies, as it can uncover neglected species, which should be included in future antivenoms. Conversely, stratification at the species level may be entirely irrelevant from a clinical perspective, if no species-specific antivenoms are available. As such, the taxonomic level at which snakes should be differentiated depends on the intended usage of the diagnostic, and for diagnostics intended to support clinicians in deciding on treatment, it will depend on the treatments available in that area. Whichever analyte is chosen, it will inform subsequent interference studies (i.e. studies that determine which, if any, substances from patient samples interfere with the measured analyte concentration). E.g., if the diagnostic measures the concentration of a cobra cytotoxin in order to diagnose a patient with cytotoxic cobra envenoming, it should be investigated whether the diagnostic also reacts with cytotoxins from the venoms of other snakes found in the same area. Additionally, in this example it should be tested whether antibiotics (which are sometimes administered to fight infections at the bitesite), prophylactically administered antivenom, or other factors found in the sample matrix (e.g. anti-idiotypic antibodies as mentioned above or medications used to treat preexisting morbidities) can interfere with analyte measurement, e.g. by potentiating the enzymatic activities of snake venom toxins (269). Several of the studies listed in **Table 1** describe investigations of the potential for cross-reactivity with other snake venoms, with some studies having screened multiple venoms, and others only a few, but none of the studies report a broader, systematic screening for interferents.

Ultimately, the design of a novel diagnostic will be fraught with compromises, as developers will have to weigh the pros and cons of diagnostic technologies for different applications. Comparatively slow and sensitive ELISAs may be ideal for coroners and researchers attempting to retrospectively identify the type of venom that caused a patient’s death, while rapid and user-friendly, albeit potentially less sensitive, LFAs may be preferable to first-responders trying to decide on appropriate first aid (or maybe, in the future, to decide on whether to use first-line-of-defense drugs, such as varespladib or batimastat). The most desirable properties of a diagnostic will thus always be determined by its intended usage, and it is unlikely that there will be a one-size-fits-all solution to developing snake venom diagnostics. Rather, multiple technologies are likely to find use in various applications.

Novel diagnostic tools for snakebite envenoming and snake identification do not have to come in the form of bioassays. Recently, an alternative was proposed with the suggestion that apps capable of recognizing photos of biting snakes and/or matching syndromes to snakes could empower healthcare providers and facilitate better treatment (212). Additionally, improved diagnosis of snakebite victims will likely depend on other initiatives in addition to novel diagnostics. For instance, studies from multiple different countries indicate that misidentification of snakes occurs, and in some cases prompts inadequate treatment (112, 115, 170, 270–273). Several authorities have therefore indicated the need for improved education and training of healthcare workers (7, 9, 244, 252, 274–277), a sentiment that is echoed in the World Health Organization’s 2019 strategy for snakebite envenoming (211).

## Conclusion

Snakebite envenoming has long been neglected, and the lack of care is – in the words of Williams et al. – a cruel anachronism (278). This neglect affects education, treatment, and diagnosis, none of which have received the attention or resources they deserve. However, with the reinstatement of snakebite envenoming on the World Health Organization’s list of top-prioritized Neglected Tropical Diseases in 2017, snakebite is garnering more attention, funding, and resources. This constitutes an excellent opportunity for scientists, physicians, and other stakeholders (many of whom have been working tirelessly for decades to alleviate the burden of snakebite envenoming) to critically revisit both current practices and new efforts within clinical snakebite management. In this relation, it is important to remember the symbiotic relationship between treatment and diagnostics. Treatments should only be administered when so indicated by differential diagnosis, and the pertinence of diagnostics depends on the available treatments. Currently, the most widely used method of diagnosis of snakebite envenoming is the syndromic approach (see **Table 3**). This approach can be highly effective, when the treating physician possesses sufficient knowledge on snakes and has been properly trained in providing correct differential diagnosis. Unfortunately, not all physicians possess this knowledge and expertise, and in some regions, utilization of the syndromic approach is challenging. If novel diagnostics could be implemented in such clinical settings, they could support the standardization of snakebite diagnosis. In combination with improved training of healthcare workers, this could in turn further improve standardization of treatment. Additional benefits could be reaped by using diagnostics to improve our knowledge of prevalence and inform the design of antivenoms and resource management.

**Table 3:** Overview of clinically commonly used diagnostic methods for snakebite envenoming in different parts of the world.

	Syndromic approach	Visual identification of the snake, if available	Patient history	20WBCT	Lab. tests	Immuno-assays
Australia	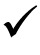	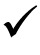	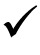	( 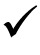 )	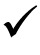	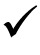
Africa	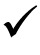	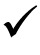	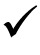	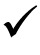	( 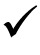 )	
Asia	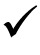	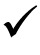	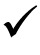	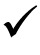	( 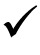 )	
Europe	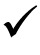	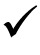	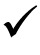		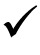	
North America	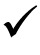	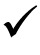	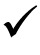	( 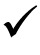 )	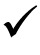	
Latin America	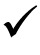	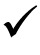	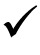	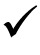	( 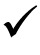 )	

Diagnostic methods can vary between and within countries in these regions, and tick marks in parentheses indicate that the method is infrequently used or only used in relatively few areas.

## Author Contributions

AL, CK, and JJ conceptualized the manuscript. AH, CK, JJ, RF, SF, and SD prepared the original draft. All authors contributed to the article and approved the submitted version.

## Funding

We thank Innovation Fund Denmark (grant 9065-00007B) for financial support.

## Conflict of Interest

CK, JJ, AH, RF, SD, and AL are shareholders in VenomAid Diagnostics ApS. CK is enrolled in an industrial PhD programme sponsored partially by the company BioPorto Diagnostics A/S, and is therefore employed by this company.

The remaining authors declare that the research was conducted in the absence of any commercial or financial relationships that could be construed as a potential conflict of interest.

The handling editor declared a past co-authorship with several of the authors, CK, JJ, and AL.
